# Clinical similarities between influenza A and B in children: a single-center study, 2017/18 season, Korea

**DOI:** 10.1186/s12887-019-1862-3

**Published:** 2019-12-03

**Authors:** Yu Na Oh, San Kim, Young Bae Choi, Sung Il Woo, Youn-Soo Hahn, Joon Kee Lee

**Affiliations:** Department of Pediatrics, Chungbuk National University Hospital, 776 1-Sunhwan-ro, Seowon-gu, Cheongju, 28644 Korea

**Keywords:** Influenza A virus, Influenza B virus, Child, Vaccination

## Abstract

**Background:**

The global burden of seasonal influenza on medical care has been one of the greatest in the pediatric population. The attention drawn to influenza B was relatively low compared to influenza A, probably because the influenza B virus was thought to be less virulent and have a lower pandemic potential. This study aimed to compare the clinical features of influenza A and B in children.

**Methods:**

This retrospective study included children diagnosed and treated for influenza as inpatients or outpatients during the 2017/18 influenza season at a tertiary referral hospital. Data regarding clinical characteristics, diagnoses, laboratory results, and vaccination histories were collected and reviewed.

**Results:**

Over the study period, 128 patients with influenza A and 109 patients with influenza B were identified. The mean age of patients with influenza B was significantly higher than that of patients with influenza A (5.6 ± 4.4 vs 4.1 ± 4.4 years, *p* = 0.010). Fever was the most common manifestation of influenza followed by respiratory symptoms. No single symptom was specifically associated with either type of influenza. The total duration of fever (4.3 ± 2.3 vs 3.7 ± 2.6 days), ‘time from fever onset to initiation of antivirals’, and ‘time from initiation of antivirals to defervescence’ were similar between the two influenza types, even though all three time periods tended to be longer for influenza B. The platelet counts and proportions of neutrophils were higher for influenza A than for influenza B infections, although the values were within normal limits for both influenza types.

**Conclusions:**

We found overall clinical similarities between influenza A and B with no less clinical significance or severity of influenza B compared to those of influenza A. Equal levels of awareness and attention should be paid to both influenza types.

## Background

The global burden of seasonal influenza on medical care has been one of the greatest in the pediatric population [1]. Influenza in children is associated with an increased frequency of outpatient visits, hospitalization, (inappropriate) antibiotic utilization, missed school days for patients and their siblings, and missed work days for patients’ parents [2].

The clinical spectrum of influenza varies depending on the child’s age, underlying disease, as well as the specific virus type [3]. As children are important vectors for the spread of the disease, vaccinating children is one of the effective means of preventing influenza [4]. In Korea, seasonal influenza vaccination has been included in the national immunization program (NIP) since the 2016/17 influenza season, starting with infants aged 6 to 11 months.

Significant antigenic mismatches between the vaccine and circulating influenza B virus strains have resulted in substantial influenza B epidemics, not limited to Korea [5, 6]. A relatively low level of attention was drawn to influenza B compared to influenza A probably because the influenza B virus was thought to be less virulent and have a lower pandemic potential [7, 8]. Recent studies have reported some similarity in the clinical characteristics of influenza A and B, with influenza B showing an occasionally higher level of severity [9, 10]. Although there are reports from Korea, there have been limited investigations regarding recent epidemics [11, 12].

This study aimed to compare the clinical features and characteristics of infections with influenza A or B during the 2017/18 epidemic season at a tertiary referral hospital.

## Methods

### Patients

This retrospective study included children treated for influenza as inpatients or outpatients at the Department of Pediatrics, Chungbuk National University Hospital (Cheongju, Korea). The epidemic period was defined as the interval characterized by a ten-fold increase or decrease in influenza tests ordered compared to that in the preceding month during the 2017/18 influenza season.

Eligibility criteria was as follows: all children aged ≤18 years diagnosed with laboratory confirmed influenza A or B during the epidemic season (September 2017–May 2018). Patients diagnosed with both influenza types were excluded for the purposes of this study. All data were collected through a detailed retrospective review of electronic medical records from the study site. Demographic data regarding the following underlying conditions were collected: asthma, chronic renal disease, atopic dermatitis, neuromuscular disease, hematologic disease, developmental delay, and preterm birth (< 37 weeks gestation). The clinical characteristics and attributes related to fever and defervescence were collected as appropriate. The laboratory data collected included the complete blood count, liver enzyme level, and C-reactive protein (CRP) level. The diagnosis of pneumonia was established either by confirming abnormalities on chest X-ray or auscultation. The vaccination records of each patient were retrieved from the vaccination registration system managed by the Korea Centers for Disease Control and Prevention.

The decision to admit a patient was made by the attending physician, independently. However, general indications for hospitalization included fever in young infants (age, 0–3 months), respiratory difficulty (including that due to community-acquired pneumonia), neurologic deficit, cardiopulmonary instability, and poor feeding. Antiviral agents were generally prescribed when positive results were obtained for either influenza virologic types. However, antiviral agents were prescribed if patients had epidemiological connections with a highly suspected influenza positive patient and had a fever, regardless of the outcomes of tests. Intravenous antiviral agents were strictly limited to patients who could not readily digest any food or had severe nausea.

### Virologic analyses

In general, fever was a major indication for virologic tests, regardless of accompanying respiratory symptoms during the epidemic season. Polymerase chain reaction (PCR) assays were performed mainly using specimens from patients on admission when the influenza antigen test result was negative despite a high level of suspicion and/or when another respiratory viral pathogen was expected.

Virus isolation from all samples was carried out using nasopharyngeal swabs. Influenza antigen tests were performed immediately after sample collection. Samples for PCR assays were stored in tubes with viral transport media at 4 °C and tests were performed within 48 h from the time of collection.

Virologic diagnoses were made using the influenza antigen test (BD Veritor™ Plus system, BD Diagnostics, Sparks, MD, USA) and/or PCR assay (Allplex™ Respiratory Panel Assays, Seegene Inc., Seoul, Korea).

### Statistical analyses

A two-sided χ^2^ test (or Fisher’s exact test when appropriate) was used to compare patient demographics, clinical outcomes, and disease severity between both types of influenza. Independent samples *t*-tests were used to examine differences between variables according to influenza type, as appropriate. Statistical analyses were performed using SPSS Statistics for Windows version 25.0 (IBM Corp., Armonk, NY, USA). All analyses with *p* values < 0.05 were considered as statistically significant.

### Ethical approval

The institutional review board of Chungbuk National University Hospital approved the study protocol (IRB no. 2018–12–010-001). The need for informed consent was waived due to the retrospective nature of the study and because the data were anonymized.

## Results

### Patient selection

**Fig. 1:**
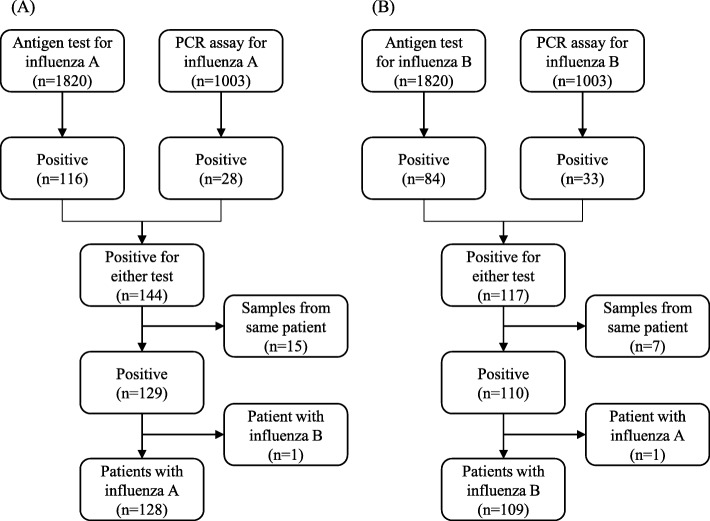
Flowchart of patient selection. **a** Patients with influenza A. **b** Patients with influenza B. PCR, polymerase chain reaction

A total of 1820 antigen tests and 1003 PCR assays were performed during the designated influenza season (Fig. 1). Using either test, influenza A or B was detected in 116/84 (*n* = 200) and 28/33 (*n* = 61) samples, respectively. Patients diagnosed with both influenza A and B via PCR assay (and with positive influenza A antigen test results) were excluded from further evaluation. Otherwise, patients with positive results for either test were included. Among patients diagnosed with influenza A and B, 23 specimens of each group were tested with both modalities. Fourteen of 22 (63.6%) PCR assay positive cases and 7 of 22 (31.8%) PCR assay positive cases tested positive using the antigen test for influenza A and B, respectively. Single cases of both influenza types showed positive results for the antigen test and negative results for the PCR assay.

### Patient characteristics

**Fig. 2:**
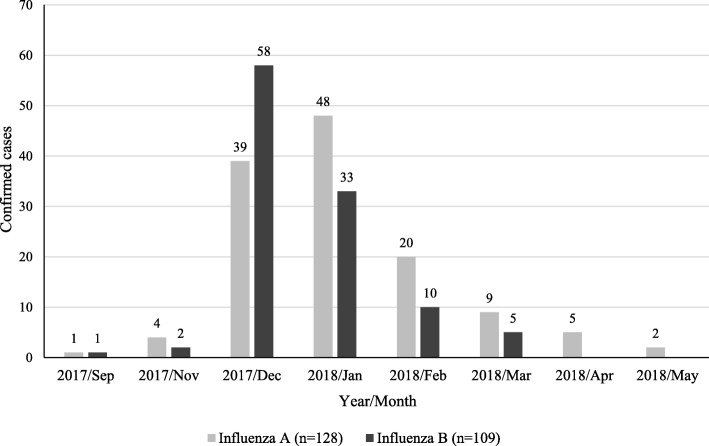
Monthly distribution of patients by influenza type

In total, 128 patients with influenza A and 109 patients with influenza B were included in the study (Table 1). A majority of the patients were diagnosed from December 2017 to February 2018 (Fig. 2). The month with the peak number of cases of influenza B (December 2017) was slightly earlier than that of influenza A (January 2018).

**Table 1:** Characteristics of patients by influenza type

Characteristics	Influenza type		*P* value
	A (*n* = 128)	B (*n* = 109)	
Boys	68 (53.1)	61 (56.0)	0.662
Age, yrs			
Mean (SD)	4.1 (4.4)	5.6 (4.4)	0.010
Median (IQR)	2.6 (1.2–4.8)	4.9 (2.1–8.0)	0.001
Age group			< 0.001
< 2 yrs	49 (38.3)	27 (24.8)	
2–5 yrs	50 (39.1)	28 (25.7)	
≥ 5 yrs	29 (22.7)	54 (49.5)	
Underlying conditions	10 (7.8)	9 (8.3)	0.900
Asthma	2 (1.6)		
Chronic renal disease		2 (1.8)	
Atopic dermatitis	2 (1.6)	1 (0.9)	
Neuromuscular disease	5 (3.9)	3 (2.8)	
Hematologic disease		1 (0.9)	
Developmental delay		1 (0.9)	
Preterm (< 37 weeks)	2 (1.6)	1 (0.9)	

Values are presented as number (%), unless otherwise specified. SD, standard deviation; IQR, interquartile range

The male to female ratios of patients with influenza A and B were 1.13:1 and 1.27:1, respectively. The mean age of the patients with influenza B was significantly higher than that of patients with influenza A (5.6 ± 4.4 vs 4.1 ± 4.4 years, *p* = 0.010). When grouped by age, the proportion of patients aged 5 years and older was significantly higher in the group with influenza B (49.5% vs 22.7%, *p* < 0.001). Underlying medical conditions were identified in approximately 8% of patients with either influenza type (7.8% vs 8.3%, *p* = 0.900).

### Comparison of symptoms between influenza A and B

Fever was the most common clinical manifestation of both influenza A and B (Table 2). There was no significant difference in the mean peak temperature for patients with both influenza types. The mean duration of fever, ‘time from fever onset to initiation of antivirals’, and ‘time from initiation of antivirals to defervescence’ were similar between the two influenza types, although those of influenza B tended to be slightly longer (Table 3).

**Table 2:** Clinical characteristics and diagnoses of patients by influenza type

Characteristics	Influenza type		*P* value
	A (*n* = 128)	B (*n* = 109)	
Fever	127 (99.2)	107 (98.2)	
Peak temperature, mean (SD)	39.4 °C (0.7)	39.3 °C (0.7)	0.323
Cough	87 (68.0)	80 (73.4)	0.361
Rhinorrhea	71 (55.5)	62 (56.9)	0.827
Sputum	38 (29.7)	37 (33.9)	0.482
Vomiting (nausea)	23 (18.0)	22 (20.2)	0.665
Seizure	20 (15.6)	13 (11.9)	0.412
Dyspnea	7 (5.5)	9 (8.3)	0.394
Diarrhea	6 (4.7)	5 (4.6)	0.971
Chill	3 (2.3)	7 (6.4)	0.193
Nasal stuffiness	1 (0.8)	2 (1.8)	0.597
Altered mentality		3 (2.8)	0.096
Rash	1 (0.8)	1 (0.9)	> 0.999
Wheezing	1 (0.8)	1 (0.9)	> 0.999
Conjunctival injection	1 (0.8)		> 0.999
≥3 yrs	*n* = 57	*n* = 66	
Sore throat	11 (19.3)	9 (13.6)	0.396
Headache	7 (12.3)	9 (13.6)	0.824
Abdominal pain	6 (10.5)	9 (13.6)	0.599
Myalgia	3 (5.3)	5 (7.6)	0.724
Arthralgia		1 (1.5)	0.460
Chest pain		1 (1.5)	0.460
Diagnosis			
Sinusitis	21 (16.4)	17 (15.6)	0.605
Pneumonia	8 (6.3)	4 (3.7)	0.367
Croup	3 (2.3)	5 (4.6)	0.476
Bronchiolitis	2 (1.6)	3 (2.8)	0.664
Lymphadenitis	2 (1.6)	2 (1.8)	> 0.999
Acute otitis media	1 (0.8)		> 0.999
Encephalitis		1 (0.9)	0.460

Values are presented as number (%), unless otherwise specified. SD, standard deviation

**Table 3:** Treatment and illness severity among patients by influenza type

Characteristics	Influenza type		*P* value
	A (*n* = 128)	B (*n* = 109)	
Hospitalization	44	35	0.783
Days of admission	*n* = 44	*n* = 34	
Mean, day (SD)	5.5 (3.2)	5.7 (2.6)	0.751
Median, days (IQR)	5 (4–6)	5 (4–7)	
Antiviral agent use	127 (99.2)	104 (95.4)	0.097
Use of IV antiviral agent	1 (0.8)	4 (3.8)	0.183
Total duration of fever	*n* = 42	*n* = 34	
Mean, days (SD)	3.7 (2.6)	4.3 (2.3)	0.269
Median, days (IQR)	3 (2–4.75)	4 (3–5)	
Fever onset to initiation of antivirals	*n* = 126	*n* = 101	
Mean, days (SD)	1.3 (1.5)	1.6 (1.7)	0.074
Median, days (IQR)	1 (0–2)	1 (1–2)	
Initiation of antivirals to defervescence	*n* = 36	*n* = 28	
Mean, days (SD)	1.4 (1.3)	1.8 (2.0)	0.305
Median, days (IQR)	1 (0.75–2)	2 (0–2)	
Antibiotic use	47 (36.7)	35 (32.1)	0.457
Supplemental O_2_ use	4 (3.1)	3 (2.8)	> 0.999
Intensive care		2 (1.8)	0.210
Ventilator care		1 (0.9)	0.460
Outcome			
Recovered without sequelae	125 (97.7)	105 (96.3)	0.706
Recovered with any sequelae		2 (1.8)	0.210
Transferred or AMA discharge	3 (2.3)	2 (1.8)	> 0.999

Values are presented as number (%), unless otherwise specified. SD, standard deviation; IQR, interquartile range; AMA, against medical advice

Respiratory symptoms were commonly observed in both influenza types. Cough, rhinorrhea, and sputum production were noticed in decreasing order. Besides respiratory symptoms, in all age groups, vomiting (nausea) was the most frequent manifestation. No patient with afebrile seizure was noted and seizure was more frequently observed among patients younger than 5 years. Among patients aged ≥3 years, sore throat was the most common complaint. None of the symptoms was significantly more common in either influenza type. Besides upper respiratory infection, sinusitis was most commonly diagnosed among patients with influenza A and B.

### Comparison of laboratory results between influenza A and B

In total, approximately three-quarters of patients with both influenza types underwent laboratory tests (Table 4). The mean white blood cell counts, hemoglobin levels, and platelet counts among patients with influenza A and B were 9527.1 ± 4382.6 vs 8592.7 ± 5198.4/μL (*p* = 0.203), 12.5 ± 1.1 vs 12.5 ± 1.3 g/dL (*p* = 0.815), and 253.9 ± 91.5 vs 227.4 ± 64.6 × 10^3^/dL (*p* = 0.032), respectively. The proportions of patients with white blood cell counts outside of the normal range for each age group were 34.1% (< 2 years), 25.8% (2–5 years), and 19.4% (≥5 years). No significant differences were shown in these proportions between influenza A and B. The proportions of neutrophils and lymphocytes among patients with influenza A and B were 63.9% vs 58.5% (*p* = 0.048) and 23.8% vs 28.2% (*p* = 0.064), respectively. There were no significant differences in the levels of aspartate transaminase, alanine transaminase, and CRP.

**Table 4:** Laboratory results of patients by influenza type

Characteristics	Influenza type		*P* value
	A (*n* = 128)	B (*n* = 109)	
Complete blood count	*n* = 93	*n* = 79	
WBC (/μL)	9527.1 (4382.6)	8592.7 (5198.4)	0.203
Hb (g/dL)	12.5 (1.1)	12.5 (1.3)	0.815
PLT (×10^3^/dL)	253.9 (91.5)	227.4 (64.6)	0.032
Neutrophils (%)	63.9 (16.8)	58.5 (19.0)	0.048
Lymphocytes (%)	23.8 (14.1)	28.2 (16.6)	0.064
Chemistry	*n* = 94	*n* = 81	
AST (IU/L)	37.1 (22.7)	39.1 (43.9)	0.705
ALT (IU/L)	20.9 (25.5)	17.9 (17.5)	0.375
CRP (md/dL)	1.7 (3.1)	1.1 (2.8)	0.218

Values are presented as mean (SD, standard deviation) value. WBC, white blood cells; Hb, hemoglobin; PLT, platelets; AST, aspartate aminotransferase; ALT, alanine aminotransferase; CRP, C-reactive protein

### Comparison of overall outcomes between influenza A and B

The hospitalization rates for influenza A and B were 34.4 and 32.1% (*p* = 0.783), respectively, with mean durations of 5.5 ± 3.2 and 5.7 ± 2.6 days (Table 3). Over 95% of patients with either influenza type received prescriptions for antiviral agents (99.2% vs 95.4%), with slightly more intravenous antiviral agents among patients with influenza B (0.8% vs 3.8%, *p* = 0.183).

Overall, 97.7 and 96.3% of patients with influenza A and B, respectively, recovered without sequelae. Two patients (1.8%) and 1 patient (0.9%) with influenza A and B, respectively, required intensive care and ventilator care, respectively. Two patients with influenza B recovered with permanent sequelae. One was a case of influenza B-associated encephalitis with a poor prognosis. This 3-year-old female infant was brought to the emergency room due to vomiting and altered mentation which started on the day of presentation. A three-day history of fever was reported; otherwise, no predisposing underlying diseases were noted. The patient was admitted to the intensive care unit and mechanical ventilation was initiated with concurrent use of intravenous neuraminidase inhibitors. Even with extensive management, this case resulted in brain death and influenza B was the only identifiable explanation for the poor clinical course. The other was a patient diagnosed with mucocutaneous lymph node syndrome (Kawasaki disease) who had a dilated coronary artery, probably as a result of the disease itself, rather than a result of influenza infection.

### Influenza vaccination status

Overall, 150 of 221 (67.9%) patients who were eligible for influenza vaccination (age ≥ 6 months) were vaccinated with either type of influenza vaccine. Of the 150 vaccinees, IIV3 (trivalent inactivated influenza vaccine) and IIV4 (quadrivalent inactivated influenza vaccine) were administered to 128 (85.3%) and 22 (14.7%) patients, respectively. The vaccination coverage rates differed among the age groups: 83.3% (50/60) among those with ages < 2 years, 88.5% (69/78) among patients with ages 2–5 years, and 37.3% (31/83) among patients with ages ≥5 years. The number of IIV3/IIV4 vaccinees and the proportion of IIV4 for each age group were as follows: 50/0 (0%) for patients with ages < 2 years, 62/7 (10.1%) for patients with ages 2–5 years, and 16/15 (48.4%) among patients with ages ≥5 years. The influenza types isolated among the patients were considered in association with the type of vaccination. Patients diagnosed with influenza prior to the vaccination were excluded. Of the 144 eligible patients, 74 (59.7%) cases of influenza A and 50 (40.3%) cases of influenza B were identified among 124 IIV3 vaccinees, while 8 (40.0%) influenza A and 12 (60.0%) influenza B cases were identified among 20 IIV4 vaccinees.

## Discussion

Influenza is a generally acute, self-limited, and uncomplicated disease in healthy children. Nevertheless, its associated morbidity and mortality rates are high among patients with underlying diseases and infants aged 2 years and younger (especially among patients aged 6 months) [13]. Influenza is frequently associated with missed school days for patients and their siblings, and missed work days for their parent(s) due to hospital visits and admissions [14].

This study identified high levels of similarity between influenza A and B, contrary to the notion that influenza A is associated with a more severe clinical course [7, 15]. There were no notable differences in the clinical characteristics, diagnoses, and severity of both influenza types. The total duration of fever, time from fever onset to the initiation of antiviral treatment, and time from the initiation of antiviral treatment to defervescence were similar between the two influenza types, in general. Nonetheless, patients with influenza B were on average 1.5 years older than patients with influenza A (*p* = 0.010), which is consistent with the reports of most studies on the epidemiology of influenza A/B, and explained by the slower accumulation of natural immunity to influenza B compared with influenza A in children [10, 16, 17].

There are mixed reports regarding the clinical characteristics of each type of influenza [16]. Consistent with the results of previous studies, fever and other respiratory symptoms were most common [16, 18, 19]. Myalgia is a more frequent symptom of influenza B [19]. However, sore throat and other subjective symptoms are also more frequently reported among patients with influenza B [16]. It is unclear whether such complaints of pain are specific to a certain type of influenza because limited studies have prospectively measured the levels of muscle-derived enzyme markers such as creatine phosphokinase as an objective finding. More common complaints of pain may be associated with increasing age among patients with influenza B than among those with influenza A.

Few studies have investigated laboratory results such as complete blood count, liver enzyme levels, and inflammatory marker levels, as these tests are routinely performed for the detection of bacterial co-infections, and differences were not anticipated [19, 20]. Total white blood cell count and CRP levels were generally normal, consistent with the findings of a previous study [19]. A finding of this study was that the platelet count and differential count of neutrophils were significantly higher in influenza A than in influenza B, although the values were within normal limits in both influenza types. When the *p* values were corrected with multiple comparisons within laboratory results with a false discovery rate of 0.25, the *p* values were 0.13 for both the platelet count and differential count of neutrophils, indicating a loss of statistical significance.

The high effectiveness of influenza vaccination has been repeatedly evaluated and remains a major means of preventing influenza [21]. As the live attenuated influenza vaccine is licensed only for use in healthy, non-pregnant persons aged 2 to 49 years, IIV3 and IIV4 are the mainstay of influenza vaccination in the pediatric population. The more frequently used IIV3 contains two strains of influenza A and only one strain of influenza B. Therefore, the two co-existing antigenically distinct lineages, B/Victoria and B/Yamagata, present a challenge. A modest lineage selection strategy that optimizes protection against influenza B using the standard trivalent vaccine may be a potentially cost-effective alternative to quadrivalent vaccines [22]. Nevertheless, if an antigenic mismatch occurs, vaccine effectiveness is decreased and marked influenza B epidemics have been repeatedly reported worldwide in those unmatched influenza seasons [5, 6].

In Korea, even after the introduction of influenza vaccinations in the NIP and widening the age window, the mainstay vaccine has been IIV3. Several influenza B mismatches were reported, with another in the 2017/18 season, the period considered in this study. The recommendations of the World Health Organization regarding the IIV3 composition for the 2017/18 season in the northern hemisphere included two influenza A strains and the influenza B/Victoria strain [23]. However, the 2017/18 season was characterized by a predominance of the influenza B/Yamagata lineage. Although the exact proportion of each influenza B type in Korea for the 2017/18 season has not been officially published yet, an almost exclusive predominance of B/Yamagata was reported [24]. This specific season was peculiar in that unlike prior influenza seasons in which epidemics of influenza A preceded those of influenza B, influenza A and B spread almost simultaneously. The peak number of patients with influenza like syndrome was identified during the first week of 2018 in Korea. This is consistent with the epidemiology shown in this study. Vaccination records showed a predominance of IIV3 among patients aged < 5 years, and almost equal proportions of IIV3 and IIV4 among those aged > 5 years. This is thought to have been influenced by the NIP, which promoted IIV3 in the younger age group. From an official report regarding the 2017/18 season, an estimated 78.9% of patients were vaccinated with IIV3, which was included in the NIP (patients aged < 5 years), while the vaccination rate increased only up to 83.5% even when IIV4 vaccinees are included [25].

In general, except for a tendency toward a longer duration of fever among influenza B patients, no significant differences were noted regarding disease severity. The hospitalization rates were approximately one-third for both influenza types with a mean duration of admission of 5–6 days. The increased tendency of time from fever onset to the initiation of antivirals may be explained by the higher mean age of patients with influenza B, consistent with the reports of previous studies [18, 19]. The increased tendency of time from the initiation of antivirals to defervescence probably originates from delayed administration and the lower susceptibility of influenza B viruses to neuraminidase inhibitors compared with influenza A viruses [26]. A slightly higher rate of prescription of intravenous neuraminidase inhibitors was noted among patients with influenza B, usually due to subjective complaints such as nausea or vomiting. Though inconsistent, neurologic complications associated with influenza B are commonly reported [27, 28]. Studies, including those focused solely on the pediatric population regarding neurologic complications, are limited, but there is a need for increased awareness and further studies are warranted.

This study had several limitations. First, even though the study site serves quite a large population in the area, the number of patients was limited, and may not represent the nation-wide population. Even though the vaccination histories of individuals were considered, as recordings for optional vaccines such as IIV4 are not compulsory, the result may not reflect the real-world vaccination situation. Besides, even though the vaccine effectiveness was not within the scope of this study, with the predominance of influenza B among older patients and with the current vaccination status which shows a high predominance of IIV4 among patients in older age groups, we posit that a much larger study population and exquisite study design are needed to identify the best choice of influenza vaccine. Despite these limitations, this study has comprehensively investigated one of the most recent epidemics, in the era of increasing awareness of influenza B. At the same time, we believe that this work will induce equal levels of attention to both types of influenza in clinical management.

## Conclusions

We found overall clinical similarities between influenza A and B with no less clinical significance or severity of influenza B compared to those of influenza A. Equal levels of awareness and attention should be paid to both influenza types.

## Data Availability

The de-identified datasets used and/or analysed during the current study are available from the corresponding author on reasonable request.

## Abbreviations

IIV3: Trivalent inactivated influenza vaccine
IIV4: Quadrivalent inactivated influenza vaccine
NIP: National immunization program
PCR: Polymerase chain reaction
